# Dynamically pre-trained deep recurrent neural networks using environmental monitoring data for predicting PM_2.5_

**DOI:** 10.1007/s00521-015-1955-3

**Published:** 2015-06-26

**Authors:** Bun Theang Ong, Komei Sugiura, Koji Zettsu

**Affiliations:** Information Services Platform Laboratory, Universal Communication Research Institute, National Institute of Information and Communications Technology, 3-5 Hikaridai, Seika-cho, Kyoto, Soraku-gun 619-0289 Japan

**Keywords:** Time series prediction, Deep learning, Pre-training, Recurrent neural networks, Elastic net, Fine particulate matter, Environmental sensor data

## Abstract

Fine particulate matter ($$\hbox {PM}_{2.5}$$) has a considerable impact on human health, the environment and climate change. It is estimated that with better predictions, US$9 billion can be saved over a 10-year period in the USA (State of the science fact sheet air quality. http://www.noaa.gov/factsheets/new, 2012). Therefore, it is crucial to keep developing models and systems that can accurately predict the concentration of major air pollutants. In this paper, our target is to predict $$\hbox {PM}_{2.5}$$ concentration in Japan using environmental monitoring data obtained from physical sensors with improved accuracy over the currently employed prediction models. To do so, we propose a deep recurrent neural network (DRNN) that is enhanced with a novel pre-training method using auto-encoder especially designed for time series prediction. Additionally, sensors selection is performed within DRNN without harming the accuracy of the predictions by taking advantage of the sparsity found in the network. The numerical experiments show that DRNN with our proposed pre-training method is superior than when using a canonical and a state-of-the-art auto-encoder training method when applied to time series prediction. The experiments confirm that when compared against the $$\hbox {PM}_{2.5}$$ prediction system VENUS (National Institute for Environmental Studies. Visual Atmospheric Environment Utility System. http://envgis5.nies.go.jp/osenyosoku/, 2014), our technique improves the accuracy of $$\hbox {PM}_{2.5}$$ concentration level predictions that are being reported in Japan.

## Introduction

Air pollution remains a serious concern and has attracted the attention of industries, governments, as well as the scientific community. One type of air pollutant that has attracted immense attention is fine particulate matter or $\hbox {PM}_{2.5}$—particles $<$2.5 $\upmu$m. $\hbox {PM}_{2.5}$ is a widespread air pollutant, consisting of a mixture of solid and liquid particles suspended in the air. Thus, $\hbox {PM}_{2.5}$ is a global issue that transcends geographical boundaries and calls for an interdisciplinary approach to solve a global problem, around which both industries and governments should play an active role. The environmental and health impacts [1, 2] of $\hbox {PM}_{2.5}$ are well documented [3–5]. Organizations and governments such as the World Health Organization [6], the USA Environmental Protection Agency (EPA) [4], UK [7], Japan [8], to mention a few, have implemented policies to support clean air in their respective towns and cities [5].

Today, most of the major air quality indexes, such as the Pollutant Standards Index (PSI) or the Air Quality Index (AQI), take into account the concentrations of $\hbox {PM}_{2.5}$ in their equations. These indexes were developed in order to provide the public with an indicator of how polluted the air is, along with the health implications that each level may imply. Very often, recommendations are also provided to the public. In December 2012, the EPA decided to strengthened its air quality standards by revising the role of $\hbox {PM}_{2.5}$ concentrations on the AQI. Concretely, the upper end of the range for the “Good” category has changed from the level of 15.0 $\upmu$g per cubic meter ($\upmu{\rm g/m}^3$) to 12.0 $\upmu{\rm g/m}^3$. This is a difference of only 3 $\upmu{\rm g/m}^3$. But in the eye of the EPA, this difference was enough to judge the previous value as not adequate to protect the public health, as required by law. Now, what is the validity of this new enforcement if the current $\hbox {PM}_{2.5}$ prediction systems are not accurate enough to make the distinction between 12 and 15 $\upmu{\rm g/m}^3$? In other words, what about the capability of the existing prediction models to meet the increasingly strict and sharp new standards?

From a governmental point of view, the costs involved due to air pollution are huge. The National Oceanic and Atmospheric Administration (NOAA) of the US Department of Commerce estimates that exposure to poor air quality is responsible for as many as 60,000 premature deaths each year and that this amount could be reduced with better predictions [9]. It is also estimated that more effective prediction methods will save US$9 billion and 64,000 jobs over a 10-year period in the USA [9]. In China, an estimated 8572 premature deaths occurred in four major Chinese cities in 2012, due to high levels of $$\hbox {PM}_{2.5}$$ pollution, and Beijing experienced a loss of US$328 million in the same year because of $$\hbox {PM}_{2.5}$$ pollution [10].

Presently, the large majority of the models being in use to address $\hbox {PM}_{2.5}$ in Japan are climate models based on Eulerian and Lagrangian grids or on Trajectory models [8]. However, an alternative to these expert models resides in artificial neural networks (NN), where high accuracy in prediction tasks has been reported [11]. In particular, a form of NN known as recurrent neural networks (RNN), in contrast with feedforward neural networks (FNN), has been shown to exhibit very good performance in modeling temporal structures [12] and has been successfully applied to many real-world problems [13]. However, it has been shown that shallow NN rapidly reach their limits due to their need for large amount of (labeled) data, which is going in contradiction with their inability to scale in complexity with the size of the network to handle the volume of data [14]. But recently, with the advent of open and big data and the alleviation of critical difficulties residing in training dense NN composed of many layers [15, 16], it has become possible to construct more complex and efficient networks. These complex networks are known as deep neural networks (DNN), and the training of such networks is often included in the appellation deep learning (DL). A review of the basic concepts of NN and DL is provided in Sect. 3.

**Fig. 1:**
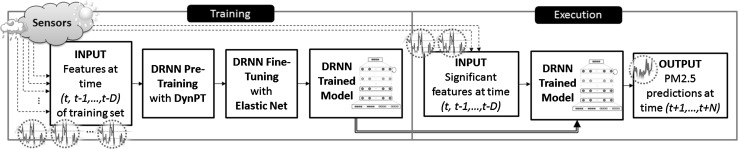
Pipeline of the proposed method. A deep recurrent neural network (DRNN) is dynamically pre-trained using a novel method called DynPT followed by fine-tuning with elastic net. The resulting trained network is then used in the execution phase to perform $\hbox {PM}_{2.5}$ predictions with less features than required during the training phase

In this work, our ultimate goal is to compute $\hbox {PM}_{2.5}$ concentration predictions in Japan using real sensor data and with improved accuracy over the currently employed prediction models. To do so, we introduce a deep RNN (DRNN) specifically designed for $\hbox {PM}_{2.5}$ prediction that is enhanced with a new pre-training method (see Fig. 1), written DynPT for convenience. DynPT improves DL techniques on the task of time series modeling, which is a field that has not received much attention yet from the DL community. Specifically, our $\hbox {PM}_{2.5}$ predictor is a DRNN that is composed of nonlinear stacked auto-encoders. The difference with conventional training of auto-encoders (AE) is that in our case, all the components of the output (or “teacher”) are not initially available, and as the training progresses, components are introduced chronologically.

We have applied our model to the case of predicting the $\hbox {PM}_{2.5}$ concentration levels of 52 cities spread all around Japan. For each city, the surrounding data come from potentially several thousands of sensors. Therefore, in an attempt to reduce the costs involved in tracking a large amount of sensors and to allow the relationship between response and predictors as understandable as possible for the scientists, variable selection is included within DRNN. As discussed in Sect. 4.3, conventional variable selection techniques suffer from major drawbacks that make their use in real-world applications unpractical. Here, we take advantage of the sparsity promoted in the internal weights of DRNN to perform sensors selection without harming the quality of the group of predictors that are selected nor the accuracy of the predictions. To do so, during the fine-tuning stage of DRNN using stochastic gradient descent, we perform regularized regression by combining the $L_1$ and $L_2$ penalties of the least absolute shrinkage and selection operator (lasso) [17] and of the ridge regression method [18], respectively. This technique is known as “elastic net” (EN) [19]. Thus, filtering the sensors is an effort toward:

- Reducing the computational and management costs that inevitably occurs when exploiting a large amount of data sources.
- Creating a physically interpretable response–predictors relationship for the end users. Those needs are often required in business-driven environments.

A detailed explanation of the proposed method is found in Sect. 4. We summarize the main contributions of this paper as follows:

- We introduce a novel pre-training method especially designed for time series prediction. The algorithm is described in Sect. 4.1, and the corresponding experimental results are discussed in Sect. 5.1.
- We propose what might be one of the first empirical research on $\hbox {PM}_{2.5}$ concentrations levels prediction that leverage the predictive power of DRNN [11, 20–23], using exclusively real sensor data, and that takes advantage of the spatial coherence in the selected sensors. Details are found in Sect. 4.2.
- We present a practical way to reduce the computational costs by filtering out sensors that do not contribute significantly to better predictions based on one of the first application of feature selection approaches in deep learning. The theoretical background is presented in Sect. 4.3, and the validity of the method is shown in Sect. 5.4.

This work extends our previous paper presented in [24] with more experimental results and with the sensors selection method. A survey of the related work is provided in Sect. 2. In the numerical experiments in Sect. 5, we compare DynPT against the canonical AE and the denoising AE, a state-of-the-art AE training method introduced in [25]. The results demonstrate the validity of our proposed approach and its adequacy to time series prediction task. Furthermore, using exactly the same set of features as and when compared to the $\hbox {PM}_{2.5}$ prediction system developed by the National Institute for Environmental Studies in Japan [26], referred to as VENUS (for Visual Atmospheric Environment Utility System) [27], our method is proven to produce more accurate $\hbox {PM}_{2.5}$ predictions. All the data used for our experiments were publicly available sensor data harvested over a 2-year period. In Sect. 6, we discuss on and clarify practical aspects related to hypotheses made in this work, and Sect. 7 concludes the study.

## Related work

To the best of the authors knowledge, this work is one of the first empirical research on $\hbox {PM}_{2.5}$ prediction with DNN using exclusively real sensor data in environmental monitoring. There exists in the literature a limited number of works that make use of DNN to predict $\hbox {PM}_{2.5}$ concentrations or time series in general. For instance, in [28] the authors propose a method for time series prediction using a deep belief network-based model composed of two restricted Boltzmann machines. Some hyper-parameters are optimized using a particle swarm optimization algorithm. In [29], several DNN architectures are presented and the efficacy of DNN for prediction tasks is further supported. A RNN is proposed in [30] to predict indoor air quality by using past information of several pollutants and other factors. These works, however, are rather an application of conventional DL methods or do not significantly improve the already known results on real-world situations.

DynPT is a novel way to train AE. Recently, a considerable amount of researchers have been studying AE. Originally, they were seen as a dimensionality reduction technique, but it has been shown that they can also be advantageously used to learn overcomplete representations of the input features. However, as AE does not learn a specific nonlinear basis, but instead a function that maps incoming data onto a high-dimensional manifold, the reconstruction error is deteriorated. This drawback is alleviated by using the regularized AE [31]. The objective is to constrain the representation in order to make it as insensitive as possible with respect to changes in input. In [32], sparse AE were introduced in the context of stacked AE to create a form of sparsity regularization. Comparing with the AE proposed in [31, 32], DynPT does not impose constraints on the input nor sparsity conditions. Actually, the weights in DynPT are intrinsically sparse initially. In [25, 33], the authors propose the denoising AE and contractive AE, respectively. In denoising AE, the task is to learn to reconstruct the input from a noisy version instead of a clean copy. In contractive AE, robust representations are learned by adding an analytic contractive penalty when minimizing the reconstruction error. The approach in DynPT might share similarities with both denoising and contractive AE in the sense that DynPT also learns from a transformed version of the reconstruction error function. However, DynPT does not resort to random noise nor additional penalty terms in the error function and is specifically intended for time series. A previous approach that supports the findings of this paper has been introduced in [34]. The authors argue that the training of deep neural networks can produce better results if the training examples are not randomly presented but organized in a meaningful order. Their experiments on shape recognition and language modeling, as well as further discussions, highlight the fact that this learning strategy can be advantageous in some particular settings.

## Problem statement and theoretical background

### Time series prediction problem

Given a set of *r* sensors *s*, we denote the set of the resulting *r* time series data by $S=\{s_1,\ldots ,s_r\}$. In this paper, we define the task of performing predictions as estimating, at a given time *t*, the value in the time series at time $(t+1,\ldots ,t+N)$ of the time series $s_z$, with $z \in [1,r]$ using the latest $(t, t-1,\ldots , t-D)$ values of $s_z \cup A$, where *A* is a subset of *S*, *N* is the prediction horizon (in hours) and *D* is the amount of past data used as input.

By using NN and a set of historical datasets, the problem consists of designing a model that can fit the inputs with the desired output. Here, the inputs are time series of past values of measured $\hbox {PM}_{2.5}$ mass concentration in Japanese cities, along with other features such as the wind speed or rain precipitations. The NN architectures that we have implemented and that we compare aim at predicting the concentration level of $\hbox {PM}_{2.5}$ several hours ahead from the current instant given historical datasets.

The estimation error of a prediction for each model is assessed using the root mean squared error (RMSE) measure defined as:

1 $${\rm RMSE} = \sqrt{\frac{\sum \nolimits _{i=1}^{N}(y_{i}-{\tilde{y}}_{i})^2}{N}},$$

where $y_i$ are the known true values of $\hbox {PM}_{2.5}$ and ${\tilde{y}}_i$ are the predictions. Throughout this paper, whenever we state that a problem is learned with good accuracy or precision, we mean that RMSE is small, if not explicitly specified otherwise.

### Neural networks and deep networks

An artificial neural network is a computational model that is composed of interconnected simple processing elements called nodes and typically organized in layers. Any pattern can be injected to the network via the input layer. The information is then processed by one or more hidden layers. There exist many different kinds of NN. Some of the most representative models are the multilayer perceptrons (MLP), the Hopfield networks and the Kohonen’s self-organizing networks [35].

Recurrent neural networks (RNN) are a class of neural networks that possess feedback connections between units, thus forming a directed cycle. This allows them to exhibit dynamic temporal behavior by using information contained in their past inputs to compute future outputs. Their high-dimensional hidden state and nonlinear behavior make them particularly suitable for integrating the information over many time steps and for expressing complex sequential relationships.

Neural network (NN) have the potential to fully take advantage of large amount of datasets to model complex nonlinear models and without the necessity to understand the intrinsic science behind the phenomenon being studied. Although this statement is true in theory, in practice shallow NN have suffered from their inability to efficiently handle very complex and huge data [15]. In response, deep networks are composed of many (vertical) layers. It has been shown that deep networks can build an improved feature space and efficiently represent highly varying functions [14]. Their recent success is often attributed to more computing power and to new training methods that take advantage of large amount of data to greedily train layer by layer the network in an unsupervised fashion, before refining the weights with usual methods in a supervised way [16].

Formally, the discrete-time dynamical system of the DRNN architecture for time series considered in this paper is written as follows [36]. Given an input ${\bf x }_i$ and an output ${\tilde{y}}_i$, where *i* represents dynamic time, we denote the hidden state of the $\psi$th layer with ${\bf h }_i^{\psi }$. DRNN with $\zeta$ layers is updated using the following equation:

2 $$\left\{ \begin{array}{ll} {\bf h }_i^{\psi } = g({\bf u }^{\intercal } {\bf x }_i) & \quad (\psi = 1),\\ {\bf h }_i^{\psi } = g({\bf d }^{\intercal }_i {\bf h }_i^{\psi -1}) &\quad (1\,<\, \psi \,<\, \zeta - 1),\\ {\bf h }_i^{\psi } = g({\bf d }^{\intercal }_i {\bf h }_i^{\psi -1} + {\bf c }^{\intercal } {\bf h }_{i-1}^{\psi }) &\quad (\psi = \zeta -1),\\ {\tilde{y}}_i = f({\bf v }^{\intercal } {\bf h }_i) &\quad (\psi = \zeta ), \end{array} \right.$$

where *g* is a nonlinear activation function, *f* is a nonlinear output function, ${\bf u }$ is the input-to-hidden weight matrix, ${\bf c }$ is the recurrent weight matrix, ${\bf d }$ represents the weight matrix from the lower layer and ${\bf v }$ is the hidden-to-output weight matrix. A common choice for the activation function *g* and the one adopted in this work is the hyperbolic tangent function. The most standard activation functions are the hyperbolic tangent (tanh) and the logistic function (sigm). Function tanh though has some advantages over sigm. The work of LeCun et al. [37] describes in detail why it is desired to have some of the properties of tanh. Additionally, other more advanced activation functions are being developed, but it is not the scope of the paper to discuss those research issues.

Auto-encoders are a particular form of MLP initially introduced to perform training via backpropagation without teacher data [38]. This is realized by setting the target output values equal to the input values. Therefore, an auto-encoder is trained to minimize the error between the input data and its reconstruction. This particularity allows them to learn automatically the features from unlabeled data in an unsupervised way. Stacked auto-encoders is a NN composed of multiple layers of auto-encoders. The outputs of each layer are fed into the inputs of the upper layer [14]. Formally, we define an encoder function *l* that aims at computing a feature vector *p* from an input *x*, such that $p=l(x;\theta )$, where $\theta =\{{\bf u }, {\bf d }, {\bf c }, {\bf v }\}$ is the set of weight parameters. Giving the dataset ${\bf x }$, we define ${\bf p }=l({\bf x }; \theta )$, where ${\bf p }$ is the “representation” obtained from ${\bf x }$. The reconstruction *q* from *p* is obtained by calling the decoder function *d*. Its role is to map the representation back into the input space with $q=d(p;\theta )$. During the training, the parameters $\theta$ for *l* and *d* are learned simultaneously and the goal is to minimize the reconstruction error:

3 $$\begin{aligned} E_{\text {AE}}(\theta )&= {} L({\bf x },d(l({\bf x }, \theta ),\theta )) \\&= {} \frac{1}{2 D M} \sum \limits _{i=1}^{D} \sum \limits _{j=1}^{M} (x_{ij}-d(l(x_{ij},\theta ),\theta ))^2, \end{aligned}$$

where *L* is a loss function defined here as the mean squared error, *D* is the number of latest past data per sensors and *M* is the number of sensors. $E_{\text {AE}}(\theta )$ is minimized by backpropagating the error and updating the parameters. In the particular case when the target output values are equal to the input values, the encoder and decoder functions reduce to the affine mappings.

## Proposed method

### Dynamic pre-training for time series

The training of deep networks was subject to many difficulties (large volume of data required, high computing power indispensable, efficient training algorithms necessary) [14, 15]. Recently, some of these drawbacks could be alleviated by performing an initial unsupervised pre-training phase that generates intermediate representations. Here, we consider the task of time series prediction and we introduce a novel pre-training principle for unsupervised learning based on the motivation that when performing multistep-ahead time series prediction [39], the intermediate representation learning does not need to follow the whole information right away at the very beginning. Instead, we argue that slowly acquiring the information and hence slowly adjusting the weights of the network along with the chronologically increasing information makes this training principle closer to what is happening physically and biologically and ultimately yields better representations for time series.

Let us introduce some notations before describing the method formally. In the proposed approach, the number of epochs is fixed. This setting is not rare in real-world applications, where the computational budget can be severely restricted. Let *H* be the maximum allowed number of epochs and *e* be the current running epoch value during the training. We also introduce the notion of number of “temporal fragmentation” or “fragments,” written $\eta$. This number represents the initial degree of separation in the components that are trained apart chronologically. An input is written ${\bf x }=\{x_1,\ldots ,x_D\}$, where *D* is the input time series length.

Then, the fragment size, or number of components contained in a fragment, is obtained with:

4 $$m = D/\eta,$$

and the epochs allocation per fragment with:

5 $$\gamma =H/\eta.$$

For convenience, we will assume that *m* and $\gamma$ are integers.

Initially, the components of the training dataset ${\bf x }$ are divided into fragments while making sure that the chronological order is preserved. The $\eta$ fragments ${\bf Z }_j$ are constructed using:

6 $${\bf Z }_j = \{x_k | k=m \times (j-1) + i; i=1,\ldots ,m\},$$

where $j=1,\ldots ,\eta$. For each fragment, a dedicated weight is created. They are designated as $w_1,\ldots ,w_{\eta }$ and belong to [0, 1] (let us note that the weights $w_j$ are not to be confounded with the weights of the neural network, which are referred to as $\theta =\{{\bf u },{\bf d },{\bf c },{\bf v }\}$).

During the training phase, the weights $w_j, j=1,\ldots ,\eta$, are updated at each epoch as follows:

7 $$w_j(e) =\left\{ \begin{array}{ll} 0 &{}\quad \big ( e \, \le \, (j-1) \gamma \big ),\\ \frac{1}{\gamma -1}(e-j \gamma ) +1 &{}\quad \big ( (j-1) \gamma \,<\, e \, \le \, j \gamma \big ),\\ 1 &{}\quad \big (e > j \gamma \big ). \end{array} \right.$$

A dummy example illustrating this algorithm is provided at the end of this section.

Afterward, the fragments are weighted and the concatenated result is stored in accordance with:

8 $${\tilde{{\bf x }}}=\{w_1 {\bf Z }_1,\ldots ,w_{\eta } {\bf Z }_{\eta }\}.$$

Finally, different from Eq. (3) for a canonical AE, the reconstruction error minimized by stochastic gradient descent for DynPT at epoch *e* becomes:

9 $$\begin{aligned} E_{\text {DynPT}}(\theta )&= {} L({\tilde{{\bf x }}}, d(l({\bf x} ,\theta ),\theta )) \\&= {} \frac{1}{2 D M} \sum \limits _{i=1}^{D} \sum \limits _{j=1}^{M} ({\tilde{x}}_{ij}-d(l({\tilde{x}}_{ij},\theta ),\theta ))^2, \end{aligned}$$

where $\theta =\{{\bf u },{\bf d },{\bf c },{\bf v }\}$ is the set of weight parameters of the network.

**Fig. 2:**
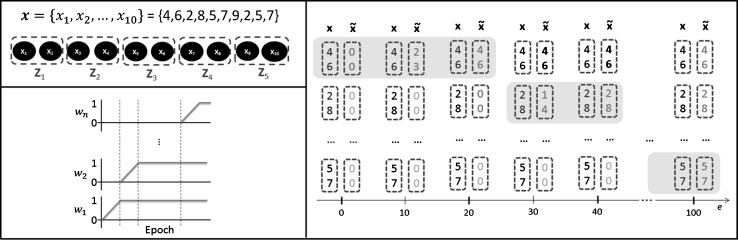
Mechanism of DynPT on a simple dummy example. The example consists of a time series having 10 time steps as an input (*D* = 10). The number of epochs is set to *H* = 100 and the number of temporal fragments to $\eta = 5$, giving a number of *m* = 2 number of time steps per fragments and the epochs allocation per fragment is $\gamma = 20$

All the hyper-parameters are chosen via cross-validation. An illustration of the mechanism of DynPT on a simple dummy example is given in Fig. 2. The example consists of a time series having 10 time steps as an input (*D* = 10). The number of epochs is set to *H* = 100 and the number of temporal fragments to $\eta = 5$, giving a number of *m* = 2 number of time steps per fragments and the epochs allocation per fragment is $\gamma = 20$. We have set ${\bf x }=\{4,6,2,8,5,7,9,2,5,7\}$. At epoch *e* = 0, before the training actually begins, the teacher data are ${\tilde{{\bf x }}}=\{0,0,\ldots ,0\}$ because all the weights $w_j, j=1,\ldots ,5$ have the null value. As the training progresses with *e*, the value of the weights increases linearly until it reached the value 1, one after another, following the weight function depicted in Fig. 2. Weights are applied to groups of time steps, i.e., the fragments. In the example, it takes 20 epochs for $w_1$ to see its value increasing from 0 to 1. Afterward, it keeps its maximal value. From epochs 21 to 40, weight $w_2$ follows its predecessor by increasing its value to 1. The process is repeated until the last epoch, where all weights eventually have their values set to the value 1.

### DRNN with heterogeneous sensor data

The set of features selected to train DRNN are the same as the ones employed by VENUS. This choice allows us to directly compare our results against VENUS. The set of features consists of hourly measured:

- $$\hbox {PM}_{2.5}$$ concentrations (PM),
- wind speed (WS),
- wind direction (WD),
- temperature (TEMP),
- illuminance (SUN),
- humidity (HUM) and
- rain (RAIN).

**Fig. 3:**
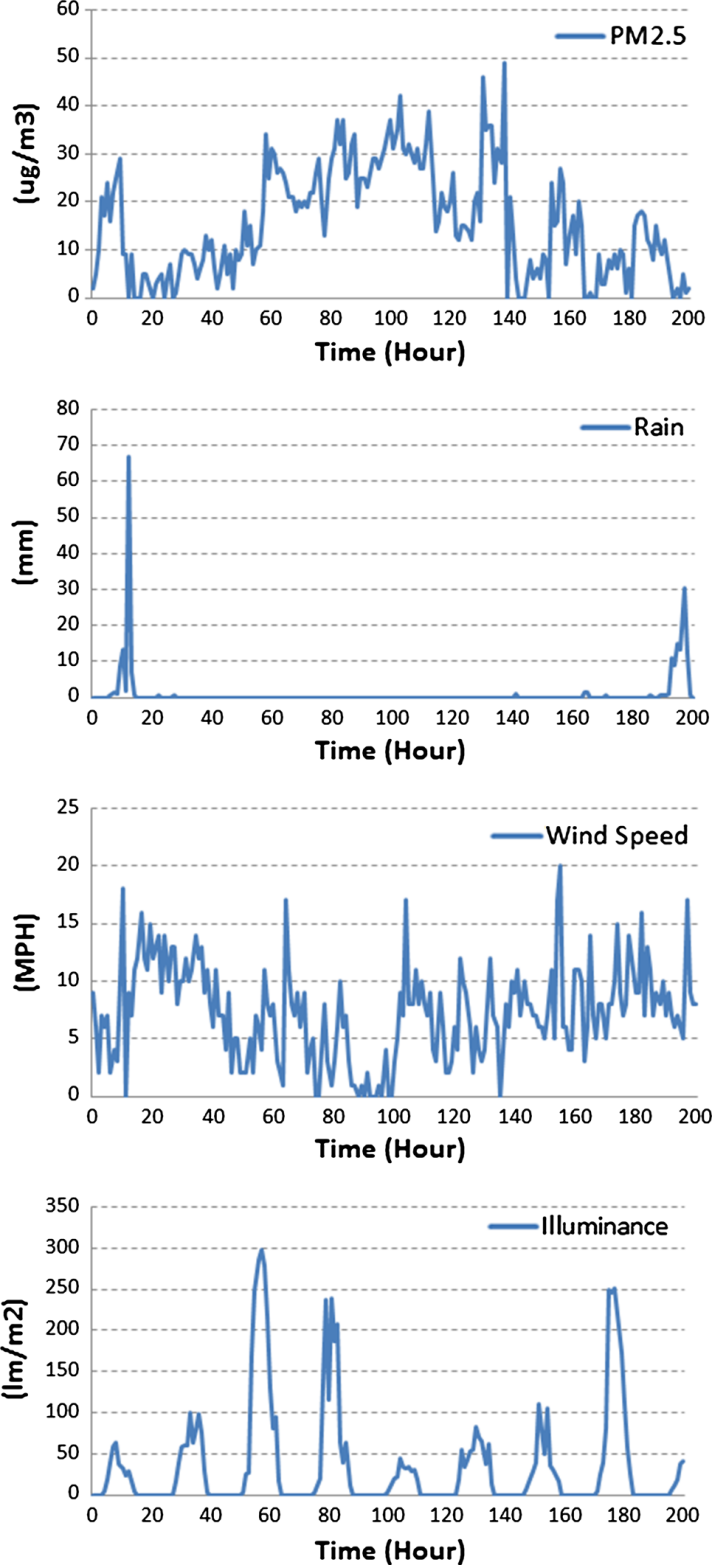
Time series plots showing characteristics of the PM, RAIN, WS and SUN data sources (from* top* to* bottom*) over a period of 200 h

We will refer to the number of features as *M*, for convenience. Six of these (PM, WS, WD, SUN, HUM and TEMP) were provided by the National Institute for Environmental Studies [26], which is a Japanese independent administrative body. The rain or precipitation data were provided by the Japan Meteorological Agency [40]. For each feature, we harvested the data of 52 cities spread all over Japan. Figure 3 depicts time series plots of some of the data sources: PM, RAIN, WS and SUN. Let us note that performing feature selection (the process of selecting a subset of relevant features) is not the main focus of this current work. Rather, our model aims at improving the predictions given standard features adopted in environmental monitoring.

The vast majority of the conventional air pollution or $\hbox {PM}_{2.5}$ concentrations levels prediction models use complex physics and chemistry. They require expert knowledge and heavily rely on parametrization. Moreover, these models do not behave well with large amount of data because of their exponential scaling with the size of the data. In contrast, NN let the data themselves build the predictor, which results in a powerful generalization ability. But as data-driven methods, NN unfortunately suffer from the illness found in the data: real-world data are indeed rarely complete and 100 % accurate. For these reasons, DRNN are an excellent match as DRNN are able to extract the useful information from the data while being robust enough to handle the noise and errors. Moreover, RNN are known for being inherently deep in time, as their hidden state is a function of all previous hidden states. This allows them learning the temporal dependencies in the data and, in particular, in $\hbox {PM}_{2.5}$ variations. More specifically, our proposed approach also takes the data of nearby cities to predict $\hbox {PM}_{2.5}$. In representation learning, this is known as the (temporal and) spatial coherence. Indeed, temporally or spatially close observations tend to lead to a small move on the surface of the high-density manifold. In the case of $\hbox {PM}_{2.5}$, those dependencies are easily observable and learned by DRNN.

For training the network, 2-year data of various features were injected. The historical data of each feature were divided into three sets: training set, validation set and testing set, having 60, 20 and 20 % of the data of each feature, respectively. We have adopted a threefold cross-validation scheme on the data and have averaged the results. The parameters that were used to train the network are reported in Table 1. All the hyper-parameters were also found via cross-validation. Different values for the hyper-parameters than those reported will most probably lead to equivalent or worse results with the datasets at hand. We have experimentally verified and reported this fact for the number of epochs in the discussion section in Sect. 6.4.

**Table 1:** Training and model parameters

Datasets span	17,545 units
Unit	1 h
Training set	60 %
Validation set	20 %
Test set	20 %
Prediction horizon (*N*)	12
Past data (*D*)	48
Number of sensors (*M*)	10
Training method	Stochastic gradient descent
Learning rate pre-training value (PT)	1e−2
Learning rate fine-tuning value (FT)	1e−3
Momentum value (CM)	0.8
Number of close cities (*K*)	3
Value for cross-validation (*k*)	3
Maximum epochs (*H*)	200
Temporal fragmentation ($\eta$)	25

**Fig. 4:**
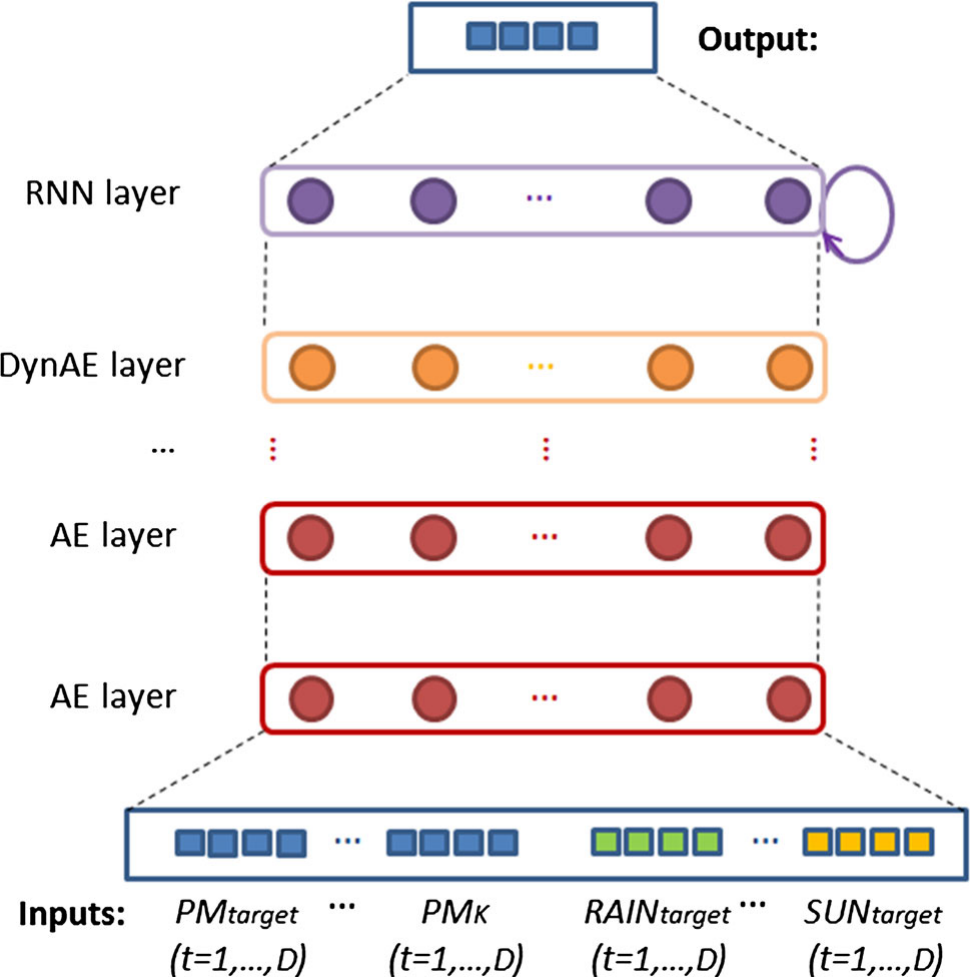
DRNN topology with *K* close cities. The input consists of *D* hours of past values of data. The resulting output is a predicted sequence of *N* values in the PM time series of the target city ($\hbox {PM}_{\rm target}$)

For each of the 52 cities for which data could be obtained, the data of all the features of a city, that we will refer to as “target” city, were injected, i.e., $\{\hbox {PM}_{\rm target}, \hbox {WS}_{\rm target}, \hbox {WD}_{\rm target}, \hbox {SUN}_{\rm target}, \hbox {HUM}_{\rm target}, \hbox {TEMP}_{\rm target}, {\rm RAIN}_{\rm target}\}$, along with $\{\hbox {PM}_{1}, \ldots , \hbox {PM}_{K}\}$ data of *K* surrounding cities that are geographically the closest capital cities from the target. Figure 4 illustrates the network topology with *K* close cities. The input consisted in *D* hours of past values of data. The resulting output is a predicted sequence of *N* values in the PM time series of the target city (see Table 1). The output is produced by a RNN layer, fed by one DynPT layer, itself above none to many AE layers.

Before using the data, preprocessing was performed to clean the datasets from known outliers. The data were also normalized to make all the input range between [0, 1].

### Sensors filtering based on sparsity

In practical applications of regression tasks, two measures are of prime importance for scientists: the accuracy of the predictions and the easiness of interpretation of the model based on the relationship between response (output) and predictors (inputs). The ordinary least squares method is known to perform poorly regarding both criteria. As an alternative, the ridge regression [18] makes use of the $L_2$ penalty and is known for achieving better accuracy. However, the model uses all the inputs, which makes the variables selection process difficult. The lasso method [17], on the other hand, employs the $L_1$ penalty on the regression coefficients, which allows much better automatic variable selection thanks to its sparse representation. In our scenario, sparse representation is an important factor to take into account. Unfortunately, lasso suffers from two major drawbacks: if there is a group of variables that are highly correlated together, lasso will arbitrarily select only one variable from the group. Also, if the number of observations is much larger than the number of predictors, then the accuracy is dominated by ridge regression [17]. To overcome these drawbacks, the elastic net was introduced in [19]. It is a regularized method that linearly combines the $L_1$ and $L_2$ penalties. It combines the advantages of both lasso and ridge regression.

Here, the elastic net method is implemented within DRNN via the stochastic gradient descent (SGD) algorithm that is used during the fine-tuning of DRNN as follows. Given the set of weight parameters $\theta =\{{\bf u },{\bf d },{\bf c },{\bf v }\}$ as defined in Sect. 3.2, SGD will minimize the regularized training error $E_{{\rm reg}}(\theta )$ given by:

10 $$E_{{\rm reg}}(\theta ) = \frac{1}{2N} \sum _{i=1}^{N}(y_{i}- {\tilde{y}}_{i})^2 + \frac{\lambda }{2}((1-\tau )|\theta | + \tau \theta ^{\top } \theta ),$$

where *N* is the prediction horizon, $\lambda > 0$ is a nonnegative hyper-parameter and $0 < \tau < 1$ is a parameter that controls the convex combination of $L_1$ and $L_2$ penalty types.

This implementation may be one of the first to successfully combine EN-based feature selection to monitoring sensors within the training of DRNN.

## Numerical results

### Dynamic pre-training DynPT

In order to assess the validity of the proposed dynamic pre-training method, we performed comparative experiments against a canonical AE and also against the widely used denoising AE [25]. Indeed, the denoising AE shares many similarities with DynPT (but DynPT is specifically intended to solve time series prediction tasks). Each case was run 10 times on the $\hbox {PM}_{2.5}$ dataset of 52 cities in Japan. The reported results are the averaged RMSE over all the runs and over all the cities. The model is a neural network initialized by stacking an AE and a basic MLP layer in order to produce 12 h predictions based on 48 h of past information. For convenience, this model will be referred to as CanAE, DenAE and DynPT, when the AE layer is a canonical AE, when the network is trained with corrupted input data and when the network is trained with the proposed dynamic process, respectively. In all cases, the network was pre-trained and fine-tuned by stochastic gradient descent.

All methods were trained on 200 epochs. The learning rate values for pre-training and fine-tuning were set equal to 1e−2 and 1e−3, respectively. In DynPT, the number of temporal fragmentation $\eta$ was set equal to 25. For DenAE, model selection was conducted for several values of corruption rate $\nu$. The reported result corresponds to the best DenAE model found for this task, which was obtained with $\nu =0.2$. This value is consistent with the typically recommended rates found in the literature.

**Fig. 5:**
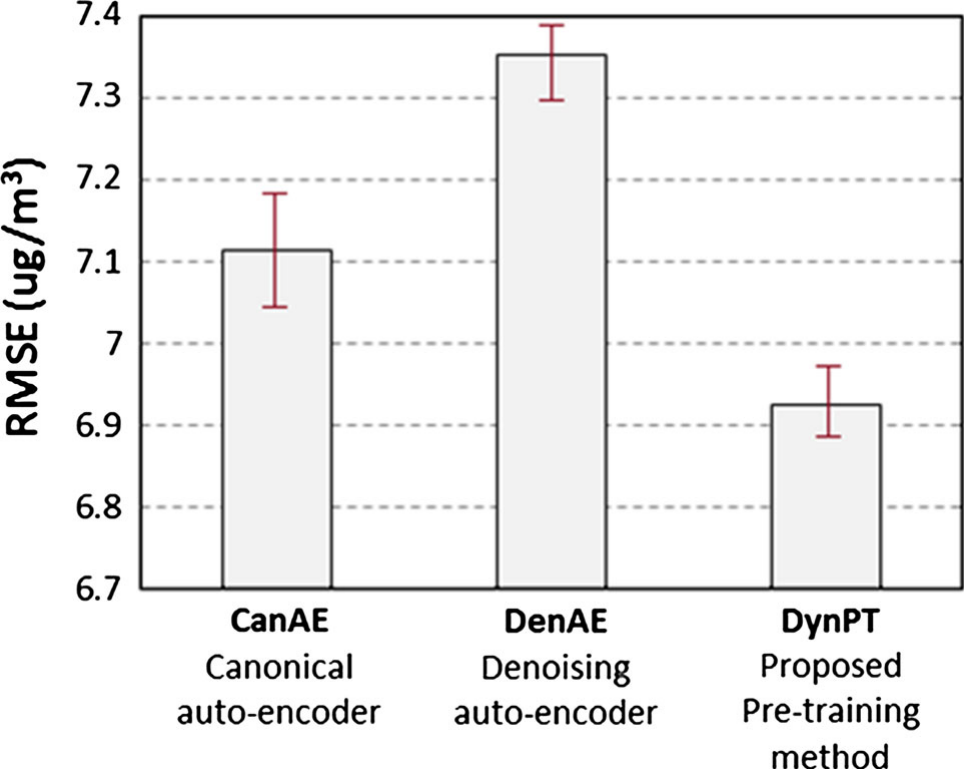
Comparison of the RMSE obtained by CanAE, DenAE and DynPT on the 12-h-ahead $\hbox {PM}_{2.5}$ prediction task for 52 Japanese cities

Figure 5 reports the results for CanAE, DenAE and DynPT. It can be observed that the best results on average in terms of RMSE were obtained by DynPT. Very interestingly, this figure also reveals an important fact: the performance of DenAE was poorer that CanAE. This observation demonstrates that although state-of-the-art AE such as DenAE achieve outstanding performance in image classification and other fields, this may not be necessarily true for other tasks such as time series prediction. The good results of DynPT demonstrate that our proposed dynamic pre-training helps achieving better predictions than a canonical AE and is also more suitable that the widely adopted denoising AE.

### DRNN for sensor data

In order to determine which NN architecture is the most suitable for $\hbox {PM}_{2.5}$ prediction with the sensor data at hand and DynPT, we have implemented four types of NN with different parameters: a feedforward NN (FNN), a fully recurrent NN (RNN), a deep feedforward NN (DFNN) and a deep recurrent NN (DRNN) (described in Sect. 4). We have considered a time step of 1 h to predict *N* = 12 h in advance with a delay of past values of *D* = 48 h. Each case was run 10 times on 52 cities in Japan. The reported results are the averaged RMSE over all the runs and over all the cities. The parameters are reported in Table 1.

**Fig. 6:**
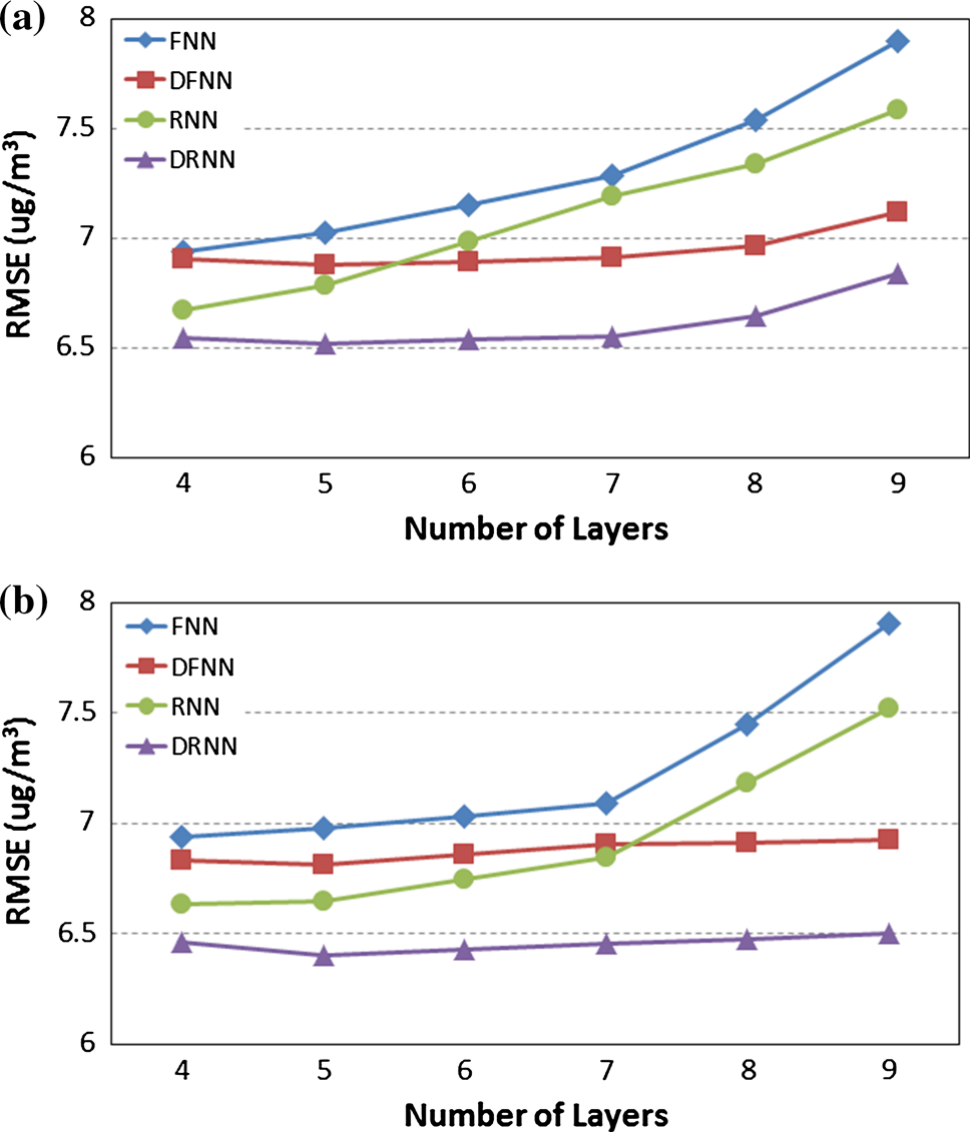
RMSE for different types and topologies of NN on the task of predicting $\hbox {PM}_{2.5}$: 4–9 layers with 30 nodes each in (**a**) and with 300 nodes each in (**b**)

The network topology of the four types of NN has ranged from 4 to 9 layers with 30 and 300 nodes for each of the layers. The number of nodes did not need to be the same for each layers, but in our experiments, this simple setting was enough to make the differences between the models clear. Figure 6a, b reports the RMSE for 30 and 300 nodes, respectively. Independently of the architecture, it can be observed that increasing the number of layers rapidly leads to overfitting. The best results were obtained with 4 or 5 layers. With our data, having 300 nodes consistently produced better results than with 30 nodes only. The numbers also validate the fact that unsupervised pre-training has a beneficial effect on the model, with DFNN and DRNN performing better than their equivalent without pre-training. Overall, the most successful architecture and topology was DRNN with 5 layers and 300 nodes each.

### DRNN against VENUS

At this point, we focus on the best model found in the previous experiment (i.e., DRNN with 5 layers and 300 nodes each) and will refer to it as DRNN in the rest of the text. We assess the performance of DRNN against the $\hbox {PM}_{2.5}$ prediction system developed by the National Institute for Environmental Studies in Japan. The system, called VENUS, for Visual Atmospheric Environment Utility System, is a regional $\hbox {PM}_{2.5}$ prediction system based on a combination of weather and chemical transport calculation. It uses numerous types of regional meteorological data, the emission data of various air pollutants and a mixture of other calculated factors.

In order to reproduce the same experimental environment, we gathered the same meteorological data as used as inputs by VENUS. The features are as follows: hourly measured $\hbox {PM}_{2.5}$ concentrations, wind speed, wind direction, temperature, illuminance, humidity and rain. Furthermore, to simulate the behavior of VENUS, we have to place ourselves in the context of a classification task. Indeed, although VENUS is able to produce exact predictions, the publicly available data that we use to perform the comparison are classified as belonging to one of six labels according to the predicted $\hbox {PM}_{2.5}$ concentration level. The classes correspond to various levels of air purity and the potential effects on human health, as with the PSI or AQI. We have extracted the predicted values of $\hbox {PM}_{2.5}$ by VENUS during the period ranging from December 2013 to February 2014, and we have compared its classification performance based on the actual values of $\hbox {PM}_{2.5}$. The classification threshold was selected as being the upper level of the “moderate air quality” level. It was straightforward to transform our initial regression task in order to get the outputs fit the classes of VENUS and simulate this classification task. By doing so, we could calculate the precision (*P*), recall (*R*) and F-measure (*F*) of VENUS during that period, that we denote as $P_{{\rm VENUS}}$, $R_{{\rm VENUS}}$ and $F_{{VENUS}}$, respectively. *P*, *R* and *F* are computed using the following equations:

11 $${\rm P}= {} \frac{t_{\rm p}}{t_{\rm p}+f_{\rm p}},$$

12 $$R= {} \frac{t_{\rm p}}{t_{\rm p}+f_{\rm n}},$$

13 $$F= {} \frac{2 P R}{P + R},$$

where the notations $t_{\rm p}, f_{\rm p}$ and $f_{\rm n}$ stand for true positives, false positives and false negatives, respectively. They compare the results of the classifier under test with the known real values of $\hbox {PM}_{2.5}$ that occurred during the concerned period. The results are reported in Table 2, which figures the performance of VENUS in its normal operation conditions (several inputs) and DRNN using past values of $\hbox {PM}_{2.5}$ only.

**Table 2:** Precision, recall and F-measure of VENUS and DRNN as classifiers

	Precision	Recall	F-measure
VENUS with multiple inputs	0.523	**0.653**	0.567
DRNN with $\hbox {PM}_{2.5}$ Data	**0.634**	0.606	**0.615**

Values in bold indicate the best performance

Those numbers are then compared with DRNN as a classifier, during the same period but using public data. The average precision, average recall and average F-measure of DRNN are, respectively, denoted as $P_{{\rm DRNN}}, R_{{\rm DRNN}}$ and $F_{{\rm DRNN}}$. It can be observed that even without any external factor, the simple use of previous historical $\hbox {PM}_{2.5}$ datasets is enough to outperform VENUS, with $F_{{\rm DRNN}} = 0.615$ against $F_{{\rm VENUS}} = 0.567$.

### Sensors selection

By using elastic net during the fine-tuning of DRNN, we could obtain a significantly parsimonious model. However, as our concern is to reduce the costs by filtering out nonsignificant sensors from the model, we define the “sensors sparsity,” abbreviated $\chi$ as being the number of sensors that have their input sparsity superior than a specified threshold. Formally, we write the sensors sparsity of sensor *a* as being $\chi _a, a=1,\ldots ,r$, where *r* is the initial number of sensors. Then, sensor *a* is considered “sparse” if the quantity:

14 $$\chi _a = \left\{ \frac{ \sum {\tilde{u}}_{ij}}{D \times \xi } \Big | {\tilde{u}}_{ij} = \left\{ \begin{array}{ll} 1 &{}\quad \big ( |u_{ij}|\le 1\hbox {e}-3 \big ),\\ 0 &{}\quad \big ( {{\rm otherwize}} \big ), \end{array}\right. \right\}$$

for $a=\{1,\ldots ,r\}, i=\{a \times D +1, \ldots , a \times D\},\,j=\{1,\ldots ,\xi \}$, is ${\ge }0.9$, where *u* is the input-to-hidden weight matrix, *D* is the number of past data used per sensors and $\xi$ is the number of nodes in the first hidden layer.

We have conducted experiments using the ridge regression, lasso method, elastic net and elastic net with sparse auto-encoders. The performance of the methods based on the RMSE and average number of filtered out sensors by the input number of sensors $\chi {/}M$ have been compared. Table 3 reports the results, along with a measure of the overall sparsity. It can be observed that as expected, the ridge regression yields better results than lasso in terms of RMSE. Although lasso considerably increased the overall weights sparsity over ridge regression, our measure “sensors sparsity” reveals that this alone is not sufficient to filter out sensors from the model. Indeed, lasso tends to select only one variable from a group of highly correlated variables, thus spreading the sparsity over all the sensors. By using elastic net, it was possible to obtain a RMSE even lower than ridge regression and a sparse network. However, the sensor sparsity level is not satisfactory in this case either. It is with the combination of sparse auto-encoders and elastic net that the best results in terms of both RMSE and sensors sparsity could be obtained. Although the overall sparsity is lower than with lasso, on average for the 52 Japanese cities considered in this work, the number of times a sensor has been found “sparse,” and thus, candidate for removal from the model for a given city was 2.1 sensors, with a maximum rejection of four sensors and a minimum of zero.

In this experiment, the value of the parameters $\lambda$ and $\tau$ have been selected using cross-validation and in such a way that the RMSE is minimized, while the sensor sparsity is maximized, simultaneously. Details are provided in Sect. 6.4.

**Table 3:** Performance of regularization methods based on RMSE and sensors sparsity

Method	Parameters	RMSE	Sparsity	$\chi / M$
Ridge (baseline)	$\lambda = 1{\rm e}{-}4, \tau =1$	6.925e−2	0.00	0.0
Lasso	$\lambda = 1{\rm e}{-}4, \tau =0$	9.450e−2	0.61	0.0
Elastic net (EN)	$\lambda = 1{\rm e}{-}4, \tau =0.9$	6.923e−2	0.06	0.0
Sparse AE + EN	$\lambda = 1{\rm e}{-}4, \tau =0.9$	**6.919e**−**2**	0.56	**0.21**

Values in bold indicate the best performance

$\lambda$ and $\tau =1$ are parameters that govern Eq. (10), $\chi$ represents the sensors sparsity and *M* is the number of sensors used as inputs of the model

## Discussion

### DRNN against autoregressive model

As the concentrations of $\hbox {PM}_{2.5}$ may not necessarily change very frequently, one may argue that the overall accuracy is rather high even with much less complex methods. To verify this hypothesis, we have compared the performance of DRNN against an autoregressive (AR) model [41]. An AR model is a representation of a type of random process that is often adopted to describe time series. It is widely used in the specialized literature to compare prediction models. The output variables of an AR model depend linearly on a number of its own previous values, known as the order of the model. We write an AR model of order *p* as AR(*p*).

The best AR model found using the same data as for DRNN was an AR model of order 6 (AR(6)). To choose the order, we have performed several experiments with candidates ranging from 1 to 10 and kept the order that provided the best results for AR. The results of the comparison against DRNN reveal that there is a considerable loss of accuracy when using AR. Indeed, the RMSE for AR(6) was of 20.8, which is around three times worse than DRNN. Therefore, the inaccuracy of AR models makes them unpractical for the prediction of $\hbox {PM}_{2.5}$. These findings are consistent with the results found in the literature and highlight the limitations of simple models over more complex methods.

### Benchmarking

To further validate our results, we have surveyed standard time series benchmarks. Among them, we have retained the benchmark known as the CATS benchmark [42]. The goal is the prediction of 100 missing values of an artificial time series with 5000 observations. The missing values are grouped in five sets of 20 successive values. Although two error criterion based on the mean squared error are proposed in [42] to compare the performance of the algorithms, only one is used for the ranking of the submissions ($E_1$), while the second criterion ($E_2$) is for additional information on the model properties. Here, we consider only $E_1$, described in [42].

We have compared DRNN against the recent work of Kuremoto et al. [28] that also proposes a method for time series prediction with a deep belief network-based model composed of two restricted Boltzmann machines (RBMs). Using the CATS benchmark and the original data, it is reported in [28] that RBMs are superior than conventional neural network models such as the MLP and the linear model ARIMA [43]. However, in the same conditions, DRNN yields even better results than RBMs, with $E_1^{{\rm DRNN}} = 1198$ for DRNN against $E_1^{{\rm RBMs}} = 1215$ for RBMs. The results are reported in Table 4.

**Table 4:** CATS benchmark results

Model	$E_1$ score
DynPT	**1198**
RBMs [28]	1215
ARIMA [43]	1216
MLP	1246

Value in bold indicates the best performance

### Fairness against VENUS

Although the data used in this paper to perform the experiments were obtained from the same agency running VENUS, it cannot be guaranteed that VENUS uses exactly the same data. However, the categories to which belong the set of features being the same, it is reasonable to claim that the comparison between the methods is fair.

Regarding the computational efficiency of DRNN against VENUS, the comparison was regrettably not possible yet. It is indeed difficult to provide absolutely fair numbers for the following reasons. First, VENUS is based on a model called Spectral Radiation-Transport Model for Aerosol Species (abbreviated as SPRINTARS) [44], which is a numerical model developed for simulating effects on the climate system and condition of atmospheric pollution by atmospheric aerosols on the global scale. $\hbox {PM}_{2.5}$ is only but one of the elements calculated by SPRINTARS. Second, the source code is not openly available and is difficult to reproduce. It can be argued, however, that as SPRINTARS requires supercomputers and that the input–output size of our proposed method is small enough to run with a standard PC while reaching comparable accuracy, the computational complexity of our proposed method is most likely largely inferior than of VENUS.

### Parameters tuning for the sensors reduction and their significance

**Fig. 7:**
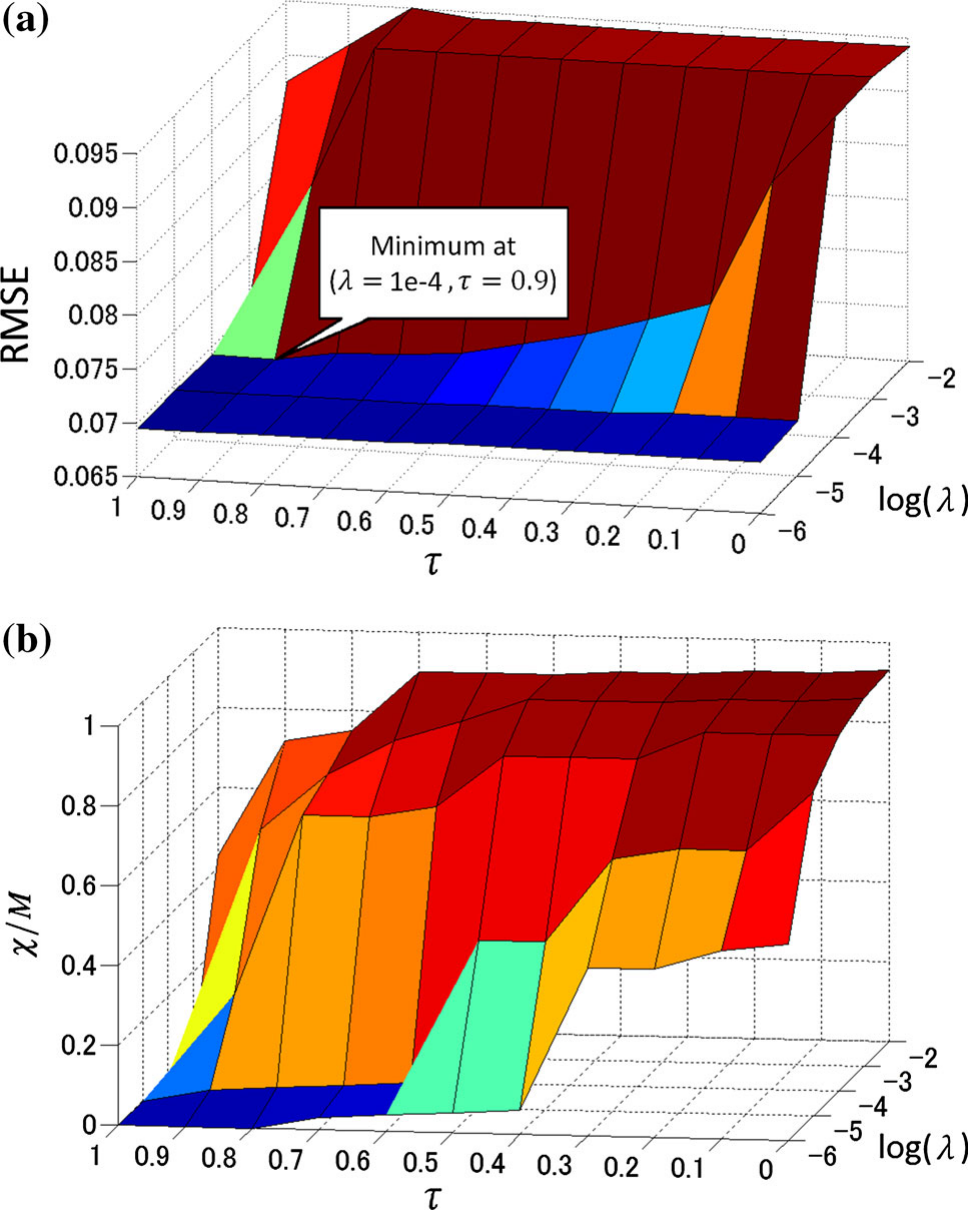
RMSE (**a**) and sensors sparsity (**b**) for values of $\lambda = \{0.01, 0.001, \ldots , 0.000001\}$ and $\tau = \{0, 0.1, \ldots , 1\}$

It was shown in Sect. 5.4 that an adequate combination of ridge and lasso regression yields better performance both in terms of RMSE and in sensors sparsity. Namely, for each city, on average slightly more than two sensors were removed from the inputs, without damaging the accuracy of the predictions. Our goal to reduce the costs involved in managing the numerous sensors was therefore reached. However, we note that the performances rely heavily on a good tuning of the parameters $\lambda$ and $\tau$. The evolution of RMSE and sensors sparsity, respectively, for values of $\lambda = \{0.01, 0.001, \ldots , 0.000001\}$ and $\tau = \{0, 0.1, \ldots , 1\}$ is plotted in Fig. 7a, b. Figure 7b shows that the higher the value of $\lambda$ is, the more the number of filtered out sensors increases. However, Fig. 7a reveals that in the extreme case where almost all but one or two sensors are removed from the model, the RMSE becomes very poor. When $\lambda$ is low, RMSE tends to reach its minimum value, at the expense of sensors sparsity that tends to reach the null value, which is not beneficial in our scenario. The minimum RMSE value that corresponds to a relative sensor sparsity ($\chi {/}M$) larger than 1 was found at coordinates ($\lambda = 1{\rm e}{-}4, \tau = 0.9$).

After filtering, a closer inspection into the significant sensors has revealed that as expected, the $\hbox {PM}_{2.5}$ data of the target city along with the surrounding cities were always considered as good predictors for the model. This demonstrates that groups of highly correlated variables did not suffer from the selection procedure. What was not expected was that the RAIN sensor was rejected around half of the time. The specialized literature provides a sound and scientifically supported reason for that phenomenon. Indeed, recent environmental studies demonstrate that the scavenging rates of fine particles that occur during rain events depend on many factors, including the size of the particles and the type of precipitations [45]. Although rain drops can actually efficiently purify the atmosphere from particles of a big enough diameter, the purification is found low in the case of very small particles such as $\hbox {PM}_{2.5}$. Actually, the concentration of $\hbox {PM}_{2.5}$ can even increase.

**Fig. 8:**
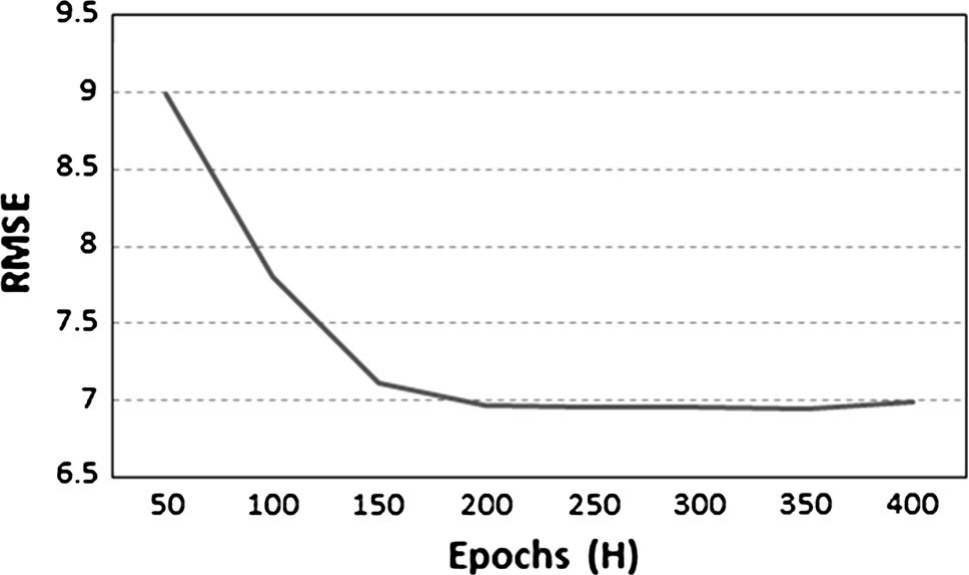
Evolution of RMSE with values for *H* ranging from 50 to 400

We also provide experimental evidences that cross-validation could find adequate hyper-parameters for the network. In Fig. 8, we report the results for the maximum number of epochs *H*. Various values for *H* ranging from 50 to 400 have been considered, and the corresponding RMSE is reported. It can be observed that the RMSE decreases sharply for small values of *H* but start stagnating after around *H* = 200, the value found automatically.

## Conclusion and future work

In this paper, we have introduced a novel pre-training method using auto-encoder especially designed for time series prediction. Our motivation is that the training of networks aiming at tackling time series forecasting tasks yield different dynamics than those relying on more static data. In light of this, we have proposed a pre-training method that allows the weights of the network to slowly adapt themselves to meet a dynamically and chronologically evolving output (teacher), which finally results in a better learning representations of the input time series. The new training method has been compared against a canonical AE and the denoising AE on the task of $\hbox {PM}_{2.5}$ prediction. The very poor performance of the Denoising AE reveals that it is not adapted to the time series prediction task. On the other hand, our method achieved higher accuracy and outperformed all the compared methods.

We have then introduced a deep recurrent neural network using this training method and that takes advantage of the spatial coherence in the several thousands of sensors from where the data are obtained. Our motivation is that our final objective is to perform $\hbox {PM}_{2.5}$ concentration predictions in Japan using exclusively real and publicly available sensor data with improved accuracy over the currently employed prediction models. The experiments revealed that our goal was reached, and the comparative experiments proved that our method could outperform the $\hbox {PM}_{2.5}$ prediction system VENUS.

Finally, we have shown that it was possible to filter out unnecessary sensors from the model by using the elastic net method. The technique could be applied effectively in deep networks as well. The groups of highly correlated sensors did not suffer from the selection, and the accuracy of the results was preserved.

For future work, we intend to further improve on the accuracy of the predictions with more advanced dynamic pre-training algorithms while at the same time performing more efficient sensors selection. The encouraging results obtained from the algorithm presented in this work may also be applied to other fields, such as health care or finance.
